# Letrozole Induced Hypercalcemia in a Patient with Breast Cancer

**DOI:** 10.1155/2014/608585

**Published:** 2014-05-15

**Authors:** Suleyman Hilmi Ipekci, Suleyman Baldane, Ercument Ozturk, Murat Araz, Huseyin Korkmaz, Fatih Colkesen, Levent Kebapcilar

**Affiliations:** ^1^Division of Endocrinology and Metabolism, Department of Internal Medicine, Faculty of Medicine, Selcuk University, 42131 Konya, Turkey; ^2^Department of Internal Medicine, Faculty of Medicine, Selcuk University, 42131 Konya, Turkey; ^3^Division of Medical Oncology, Department of Internal Medicine, Faculty of Medicine, Selcuk University, 42131 Konya, Turkey

## Abstract

Hypersecretion of PTHrP is a relatively common cause of malignancy-related hypercalcemia. However, there is only one case report of letrozole induced hypercalcemia. A 52-year-old female patient was referred to our clinic because of the recent discovery of hypercalcemia (11.0 mg/dL). The patient had a history of left breast carcinoma. She had started a course of letrozole (aromatase inhibitor; 2.5 mg dose/day) ten months earlier. Patient's parathyroid hormone-related protein levels were normal and a bone scintigram revealed no evidence of skeletal metastasis. Other potential causes of high calcium levels were ruled out. We recognized that, when letrozole was taken at one dose daily (2.5 mg), she had recurrent hypercalcemia. Our experience suggests that letrozole may precipitate hypercalcemia in a patient with breast cancer.

## 1. Introduction

Hypersecretion of PTH related peptide is a relatively common cause of malignancy-related hypercalcemia. However, there is only one case report of letrozole induced hypercalcemia. We report a case of hypercalcemia that appeared with letrozole treatment in a patient with breast cancer.

## 2. Case

A 52-year-old female patient was referred to our clinic because of the recent discovery of hypercalcemia in a routine oncological control. Her oncologist, assuming that the diagnosis was most likely to be tumor-induced hypercalcemia, referred her to an endocrinologist, who undertook further tests to reveal the etiology of hypercalcemia. The physical examination revealed her to be eupneic, blood pressure of 120/80 mmHg with 84 beats per second. The patient had a history of left breast carcinoma. Our patient had undergone a left mastectomy one year previously (estrogen-receptor positive, progesterone-receptor positive, Her2 negative) and received letrozole without any complications until hospitalization. She remained well and achieved a complete response without an increase of carcinoembryonic antigen (CEA, (normal value <5 ng/mL)) or carbohydrate antigen 15-3 (CA 15-3, normal value: 0.5–29 IU/mL) since ten months.

Currently she is 1.62 m tall, weighting 62 kg, with a body mass index (BMI) of 23 kg/m^2^ and using letrozole only for the indication prescribed. She had started a course of letrozole ten months earlier. Her family history was unremarkable. As her hypercalcemia was mild and did not elicit any symptoms, she did not require immediate measures to correct this.

Investigations to determine the cause of the hypercalcemia were performed. Initial laboratory evaluation showed hypercalcemia at 11 mg/dL, iPTH as 15 pg/mL normal albumin 4.3 g/dL, and phosphorus level 3.5 mg/dL (Normal: 2.5–4.8) and creatinine 0.57 mg/dL. 24 h urinary calcium excretion was found to be 247 mg/day (normal: 100–300 mg/day). Further blood tests demonstrated that alkaline phosphatase level was 64 U/L (35–104) and 25-hydroxy vitamin D level was 22 ng/mL (normal: 20–30) within normal limits. A dual energy X-ray absorptiometry (DEXA) scan was performed, which revealed mild osteopenia at the left femoral neck (T score: −1.6). No previous bone mineral density (BMD) measurement had been taken prior to this.

**Figure 1:**
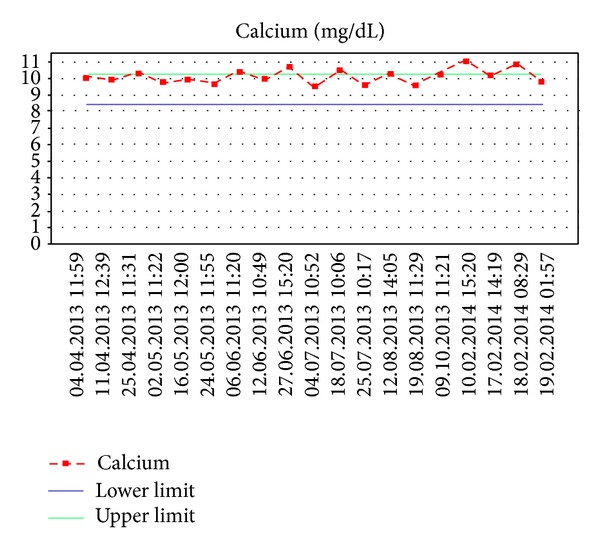
Serum calcium concentrations showing temporary remission of hypercalcemia.

**Figure 2:**
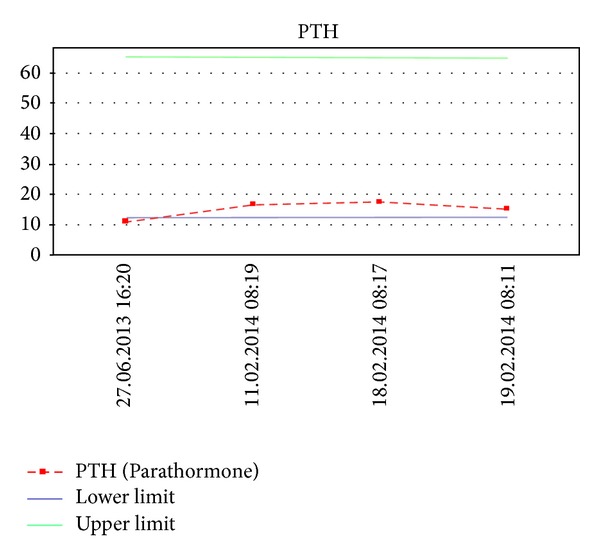
Course of serum parathyroid hormone concentrations.

On other days, our patient's serum calcium and iPTH level returned to within normal range without any medications. When we analyzed the sequence of laboratory tests, there were episodes of calcium and iPTH levels with fluctuating on repeat analyses over a period of ten months between 9.5–11 mg/dL (Figure 1, normal: 8.5–10.2 mg/dL) and 11–17 pg/mL (Figure 2, normal: 12–65), respectively.

A workup investigation to exclude other causes of hypercalcemia was done. She underwent an extensive evaluation including careful history, family history, physical examination, and lab work to exclude possible other causes of hypercalcemia. Parathyroid hormone related peptide was 0.28 pmol/L (normal: 0–1.3) and bone scan was normal, thus making malignancy-related hypercalcemia unlikely. Computed tomography of thorax, neck and abdomen, and PET-BT did not reveal any malignancy.

Although serum phosphorus was normal and iPTH was not increased, in order to rule out hyperparathyroidism, evaluation of the parathyroid glands was performed using ultrasound and MIBI scan, which were normal. Clinically, patients with FHH (familial hypocalciuric hypercalcemia) have relative hypocalciuria and inappropriately normal or elevated iPTH in the face of persistent mild hypercalcemia, none of which our patient had.

In addition to malignancy-related hypercalcemia, the differential diagnosis of hypercalcemia includes calcium supplements hypervitaminosis D, milk-alkali syndrome, granulomatous diseases, medications, inflammatory and rheumatic diseases, and other endocrine disorders.

25-hydroxy vitamin D level was not elevated ruling out hypervitaminosis D. Tuberculin test, computed tomography of thorax, and bone survey were normal, thus ruling out a granulomatous process (tuberculosis or sarcoidosis). Erythrocyte sedimentation rate and ANA level were normal, ruling out inflammatory and rheumatic diseases. Thyroid-stimulating hormone and free thyroid hormones should be checked to help rule out hyperthyroidism. Free T_4_, Free T_3_, and TSH were normal, ruling out hyperthyroidism, and there were no signs or symptoms suggestive of Cushing disease, adrenal insufficiency, acromegaly, or pheochromocytoma. Normal prolactin level and IGF-I in the age- and gender-matched normal range excluded the diagnosis of prolactinoma and acromegaly in our patient, respectively. Basal cortisol level was 19 *μ*g/dL, and therefore we could rule out adrenal insufficiency. An overnight dexamethasone suppression test (DST) was done. After a 1-mg dose DST, the plasma cortisol level was 0.8 *μ*g/dL. Hence, we could rule out Cushing syndrome. A 24-hour total urinary metanephrines and fractionated catecholamines were within normal range. She had no evidence of leukemia and lymphoma and a normal complete blood count. Serum electrophoresis was negative for monoclonal proteins. She indicated no symptoms of peptic ulcer, and there was neither exogenous vitamin D intake nor family history of endocrinopathy. She had no history of taking vitamin A, calcium supplements, and thiazide medication and no recent history of immobilization. Assessment of bone metabolism using markers of bone turnover could yield useful information and guide management decisions in our case. Urinary hydroxyproline level was significantly increased (35 mg/24 h/m^2^; normal: 22–55 years—8.5–23.5 mg/24 h/m^2^).

Because of sufficient vitamin D level, oral bisphosphonate was administered and letrozole was restarted at one-half dose (1.25 mg). She was discharged and we closely follow up patient's serum calcium, CEA, CA 15-3, and for distant metastasis.

## 3. Discussion

Intensive investigations did not lead to any underlying cause for elevated serum calcium and urinary hydroxyproline level, and hypercalcemia may be explained by using letrozole treatment in our patient.

Malignancy-related hypercalcemia is due to increased osteoclast activities and bone resorption caused by the increased release of various mediators from the tumor or nontumoral host cells [1–4].

Aromatase inhibitors (AIs) are an important component of adjuvant therapy in postmenopausal women with estrogen receptor positive breast cancer. Letrozole works by blocking aromatase that converts androgens to estrogen [5]. While the main source of estrogen in premenopausal women is the ovaries, the main source in postmenopausal women is the adrenals. Adrenal androgens are converted to estrogens by aromatase enzymes. Aromatase inhibitors prevent this conversion. In postmenopausal women, AIs cause relatively rapid decreases in circulating estrogen. Treatment with AIs, therefore, results in bone loss due to estrogen deficiency [6]. Estrogens exert a major effect in women on bone remodeling by inhibiting interleukin-6 (IL-6) productions that reduces bone resorption and also controls the timing of osteoclast apoptosis. Estrogens deficiency, therefore, results in a longer life span of osteoclasts [6]. Urinary hydroxyproline level was significantly increased in our patient. Hydroxyproline is the major breakdown product of collagen, the primary protein of the bone matrix. It is considered as clinical index of bone resorption and a major determinant of bone status. During bone loss, collagen fibrils are broken down and hydroxyproline is excreted in the urine [7].

Serum intact PTH and phosphor were not low in our patient but they were lower limits of the reference ranges. We considered they might be mild hypercalcemia and mild deficiency of vitamin D. Changes in circulating calcium concentrations alter PTH secretion via a negative feedback system. An increase in calcium binding stimulates phospholipase C and inhibits adenylate cyclase and the resultant rise in phosphatidylinositol trisphosphate and reduction in cAMP concentrations reduces PTH release [8]. As a result, PTH secretion is inversely proportional to serum calcium concentration [8]. In addition to this negative feedback control of PTH secretion, a normal in vitamin D concentration reduces PTH level. A serum 25-hydroxy vitamin D of 40 nmol/L (40 nmol/L = 16 ng/mL) appears to represent a critical point in vitamin D metabolism at which vitamin D deficiency begins to unduly stress normal calcium homeostasis. We showed that serum 25-hydroxy vitamin D concentrations <40 nmol/L (25-hydroxy vitamin D level was 22 ng/mL in our case) are associated with significantly higher serum PTH [9]. However, the secretion of PTH is never fully suppressed like in our case.

On the other hand, the most common cancers associated with hypercalcemia are breast and lung cancer and multiple myeloma. There are three major mechanisms [10–12] by which hypercalcemia of malignancy can occur: osteolytic metastases with local release of cytokines (including osteoclast activating factors); tumor secretion of parathyroid hormone-related protein (PTHrP); and tumor production of 1,25-dihydroxyvitamin D (calcitriol). Patient's parathyroid hormone-related protein levels were normal, thus making PTHrP related hypercalcemia unlikely. One of the limitations of this report is that cytokines such as IL-6, IL-8, IL-1, and VEGF which are secreted by breast cancer cells were not determined and may contribute to the effects of PTHrP on bone resorption [13]. Increased production of 1,25-dihydroxyvitamin D (calcitriol) is the cause of almost all cases of hypercalcemia in Hodgkin lymphoma and approximately one-third of cases in non-Hodgkin lymphoma [11]. Although 1,25-dihydroxyvitamin D-induced hypercalcemia has not been described in patients with breast cancer, we should have measured the 1,25-dihydroxyvitamin D level which was the second limitation of our report.

Clinical trials have found that the AIs, unlike the hormone therapy tamoxifen, increase bone fracture risk [14]. Moreover, flare hypercalcemia has a rapid onset that characteristically occurs within several days of starting therapy; symptoms are usually short-lived and serum calcium levels return to normal when the offering agent is withdrawn [4]. However, the temporal relation of the onset of hypercalcemia and administration of letrozole may suggest a possible role for the drug.

Women receiving adjuvant AIs therapy for breast cancer are at increased risk for fractures [14], which has been shown to result in increased morbidity and mortality in women with postmenopausal osteoporosis [15]. We recommended that patients should be initiated on bisphosphonate therapy for the prevention and treatment of osteopenia and flare hypercalcemia. For women who have osteoporosis and are on aromatase inhibitors, bisphosphonates should help reduce fracture risk [16]. The therapy contains a potent nitrogen containing drug, that is, alendronate, which binds hydroxyapatite crystals of bone with high affinity and inhibit bone resorption by decreasing osteoclastic activity and its growth. After the inhibition of resorption, these agents become affixed to the bone matrix, where they reside until the remodeling begins again [17]. Reduction in bone loss during antiresorptive treatment is demonstrated by decreased excretion of hydroxyproline after antiresorptive therapy [18].

We report a case of hypercalcemia that occurred after initiation of letrozole in a patient with breast cancer. To our knowledge, the association of hypercalcemia and letrozole has been previously reported one time [4]. If this occurs, letrozole might be restarted cautiously with therapeutic benefit.
